# Breaking barriers: Enhancing access to dementia clinical trials in the United Kingdom—Insights from the Scientific Advisory Board of the Dame Barbara Windsor Dementia Goals Programme

**DOI:** 10.1002/alz.71621

**Published:** 2026-07-05

**Authors:** Ruth M. McKernan, James B. Rowe, Sally John, Eric Karran, Helene A. Fachim, Sara Imarisio, Raj Long, Charlotte Teunissen, Sarah P. Slight, Catherine Mummery, John Hardy

**Affiliations:** ^1^ Chair of the Scientific Advisory Board of the Dementia Goals Programme Operating Partner of SV Health Investors London UK; ^2^ Department of Clinical Neurosciences and Cambridge University Hospitals NHS Trust University of Cambridge Cambridge UK; ^3^ Informatics & Predictive Sciences Bristol Myers Squibb Cambridge Massachusetts USA; ^4^ University College London London UK; ^5^ Vannin Neuropharma Consulting Ltd Canterbury UK; ^6^ Medicines Discovery Catapult Macclesfield UK; ^7^ Senior Advisor Consultant Gates Ventures Kirkland USA; ^8^ Department of Laboratory Medicine Neurochemistry Laboratory Amsterdam UMC Amsterdam the Netherlands; ^9^ School of Pharmacy Newcastle University Newcastle upon Tyne UK; ^10^ Dementia Research Centre Department of Neurodegenerative Disease UCL Queen Square Institute of Neurology University College London London UK; ^11^ Deputy‐chair of the Scientific Advisory Board of the Dementia Goals Programme Department of Neurodegenerative Disease UK Dementia Research Institute Centre at University College London London UK

**Keywords:** clinical trials, cohorts, pharma, registry

## Abstract

The Dame Barbara Windsor Dementia Goals Programme was launched by the UK Government to accelerate the development and delivery of new treatments for dementia. We present the recommendations from the Scientific Advisory Board, to enable timely access to therapies for the wider population, reducing health system burden while improving patient outcomes. The recommendations focus on three areas: (i) establishing a new dynamic national patient registry for clinical trial recruitment; (ii) the use of biomarkers to improve early and accurate diagnosis; and (iii) a framework for end‐to‐end implementation across the landscape of healthcare, research and regulators. A *Brain Aging Registry for Biomarkers, Access to trials, Research and Adoption* would support recruitment, monitoring, and personalized care. Embedding digital and biomarker innovations into routine care would improve personalized and equitable dementia services, with earlier diagnosis and more effective prevention. Robust patient and public involvement is required, to ensure transparency, trustworthiness, and meaningful participation.

The Dame Barbara Windsor Dementia Goals Programme^1^ was launched by the UK Government in August 2022 to accelerate the development and delivery of new treatments for dementia diseases and other neurodegenerative conditions. The heart of this ambition is a vision to enable timely access to therapies for the wider population, reducing health system burden while improving patient outcomes. The Programme is structured around three pillars:

- Pillar 1: Biomarkers and experimental medicine—discover, validate and operationalize a suite of clinically actionable and decision‐enabling biomarkers. 
- Pillar 2: Clinical trial innovation—establish research‐ready infrastructure for accelerated trial initiation and increased patient participation. 
- Pillar 3: End‐to‐end implementation—align translational research, clinical practice, and regulatory frameworks to prepare health systems.

To support this ambition, a Scientific Advisory Board (SAB) was convened, drawing on expertise from industry, academia, regulatory bodies, and clinical practice. The SAB's recommendations focus on three interdependent areas: establishing a dynamic national patient registry to support clinical trial recruitment, advancing biomarkers to enable early and accurate diagnosis, and developing a framework for end‐to‐end implementation across the NHS and research landscape.

The SAB strongly recommends the creation of **BARBARA**—the ***B*** *rain* ***A*** *ging* ***R*** *egistry for* ***B*** *iomarkers*, ***A*** *ccess to trials*, ***R*** *esearch and* ***A*** *doption*— a virtual, nationwide, dynamic infrastructure that integrates existing registries and cohorts with new biomarker data. BARBARA will support recruitment across the disease continuum, from at‐risk individuals to those already diagnosed, and provide a powerful national asset for clinical trials, real‐world data collection, and future treatment evaluation.

At the core of this approach is the adoption of pTau217, a highly validated plasma biomarker of Alzheimer's pathology, recently approved by United States Food and Drug Administration (FDA) and now used in memory clinics internationally, including in the United Kingdom, combined with digital cognitive assessments to enable pre‐screening and monitoring. This dual approach is envisaged to offer a scalable, patient‐friendly alternative to more invasive procedures and should improve the identification of people at risk, support regulatory alignment, and reduce trial failure rates for sponsors running clinical trials.

Implementation of BARBARA should proceed in a phased, pragmatic manner, including biomarker validation, developing the registry with appropriate governance structures, patient engagement, and regulatory dialogue. The success of this approach depends on trust, inclusivity, and patient involvement at every stage. With the right foundation, the United Kingdom can lead globally in dementia trial readiness and equitable access to emerging therapies.

Progress is already underway, with several national initiatives laying the groundwork for BARBARA. Through Innovate UK, four Small and Medium‐sized Enterprises (SMEs) are contributing to the global Bio‐Hermes 2^2^ project, comparing low‐cost assessments with brain imaging across diverse populations. In parallel, programs like READ‐OUT^3^ and the UK Dementia Trials Network (UKDTN) are piloting biomarker integration into NHS settings. READ‐OUT could serve as a route for patient inclusion into the registry, alongside recently phenotyped individuals from population cohorts such as Our Future Health (OFH) and UK Biobank. The Medical Research Council (MRC) ‐funded Dementia Trials Accelerator (DTA) is also expanding access and awareness through community engagement, aiming to scale trial participation from under 100 in 2021‐22 to tens of thousands. Crucially, these efforts are being guided by robust patient and public involvement (PPI) to ensure transparency, trust, and meaningful participation.

## 1 BACKGROUND

Dementia is the leading cause of death in England and already costs the UK Government around £34.7 billion annually, a figure projected to rise to over £94 billion by 2040.^1^ Recent advances have demonstrated that, like cancer, dementia is a treatable disorder with the promise of many more specific therapeutics in the future. However, major challenges persist in diagnosis, equitable access to treatment, and the under‐representation of diverse populations in clinical trials.

Around 70% of people with dementia are not currently accessing treatment or trials, often due to regional variation in service delivery and ongoing stigma.^4^ While amyloid and Tau remain the most studied pathological targets, dementia comprises multiple neurodegenerative processes, requiring precise diagnosis for optimal therapy matching in the future.

A core barrier to Alzheimer's trial recruitment has been the low success rate in identifying eligible patients, largely reliant on costly and invasive PET imaging, and patients having declined beyond eligibility for early intervention studies. Emerging blood‐based biomarkers (BBMs) such as pTau217 now offer a scalable alternative for early detection and pre‐screening, enabling broader access across primary care and underserved communities. While BBMs indicate risk, not clinical diagnosis, monitoring of pTau217 can identify patients in disease progression and accelerate trial recruitment more equitably and efficiently.

## 2 CLINICAL TRIALS IN NEURODEGENERATION IN THE UNITED KINGDOM

Globally, the Alzheimer's disease (AD) pipeline includes 138 drugs across 182 clinical trials,^5^ with additional candidates in development for non‐AD dementias. Yet the United Kingdom has contributed minimally to this effort: between 2021 and 2022, only 61 individuals with Alzheimer's dementia participated in late‐stage, commercially sponsored trials in the United Kingdom.^6^

This underrepresentation reflects longstanding systemic barriers. The UK healthcare system has not been configured to support large‐scale dementia trial delivery, particularly in early identification, biomarker access, and participant pre‐screening. Diagnostic pathways are not aligned with trial eligibility, and recruitment infrastructure (e.g., registries, digital tools, biomarker platforms) has been fragmented, focused on research and understanding the disease. Research and clinical services have also operated in silos, with slow contracting processes between sponsors and National Health Service (NHS) institutions further limiting trial readiness.^7^

**FIGURE 1:**
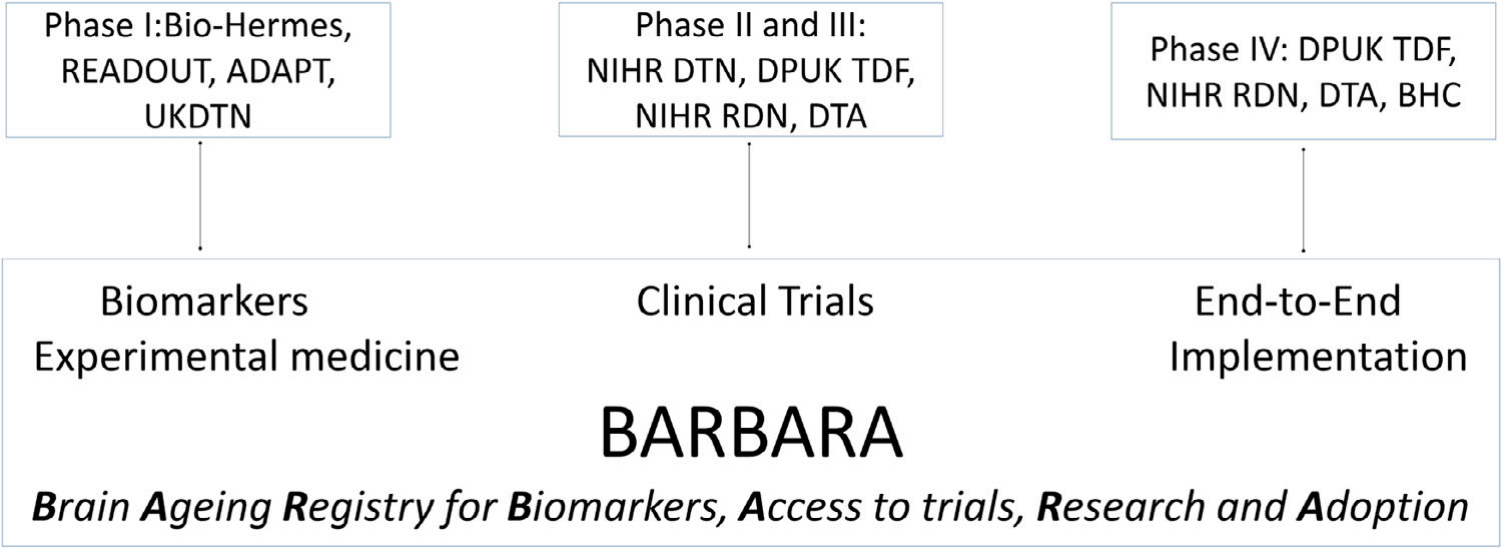
Overview of BARBARA and the integrative platform for the Dementia Goals Programme. BARBARA will act as the central vehicle to consolidate and deliver the three strategic pillars of the Dementia Goals Programme: Biomarkers and experimental medicine, clinical trials, and end‐to‐end implementation. By providing a unified infrastructure for data integration, sample access, participant identification, and longitudinal follow‐up, BARBARA enables alignment across these pillars, supporting translational research, accelerating trial readiness, and enabling real‐world deployment of innovations.

While the United Kingdom has been better represented in early‐phase studies,^8^ the overall ecosystem has lacked coordination. However, recent investments, including the formation of the UK Dementia Trials Network (UKDTN) and the Dementia Trials Accelerator (DTA) along with biomarker validation pilots are improving readiness for clinical studies and multiple entities are contributing more to all phases of clinical trials (see Figure 1). More still needs to be done to match the efficiency of starting clinical trials in some other countries e.g Spain and the United States.

## 3 THE CASE FOR A SINGLE NATIONWIDE DYNAMIC VIRTUAL REGISTRY: BARBARA

The current global landscape of dementia registries and cohorts is highly fragmented, with many operating at local or national levels without standardized frameworks or comprehensive longitudinal real‐world data (RWD) collection. This lack of cohesion hinders research progress and impedes the development of effective patient care strategies.^8^ Numerous national and international initiatives exist (See Appendix S1) and many registries are disease‐specific or restricted to closed, non‐recallable studies. While these have advanced research notably, they are not designed for large‐scale trial recruitment or long‐term integration with real‐world clinical care. Gaps in standardization, interoperability, and access limit their current utility.

To address this, the Scientific Advisory Board (SAB) proposes the creation of BARBARA — a virtual, dynamic, nationwide registry for dementia. The terminology registry reflects the intended role of BARBARA as a national resource to support recruitment, monitoring, and personalized care, without duplicating existing data custodianship. BARBARA will not replace existing registries, but rather integrate them into a single infrastructure, as far as possible, enabling cross‐cohort data harmonization and participant recontact. BARBARA is defined as virtual, dynamic, and nationwide: *Virtual*, as it will build on and link pre‐existing registries and cohorts, supporting remote participation and integration with health data systems. *Dynamic*, because it will be continuously updated with new participants, and because participants’ data can be updated with new biomarker data (e.g. blood‐based or digital tools), enable long‐term follow‐up of disease progression. *Nationwide*, ensuring broad demographic and geographic inclusion to improve access to clinical trials across the United Kingdom and create greater combined value than isolated data collections. The initial focus on AD reflects the maturity of AD‐specific biomarkers. However, with the emergence of specific diagnostic biomarkers for non‐AD dementias, BARBARA will broaden its scope to facilitate non‐AD recruitment.

**FIGURE 2:**
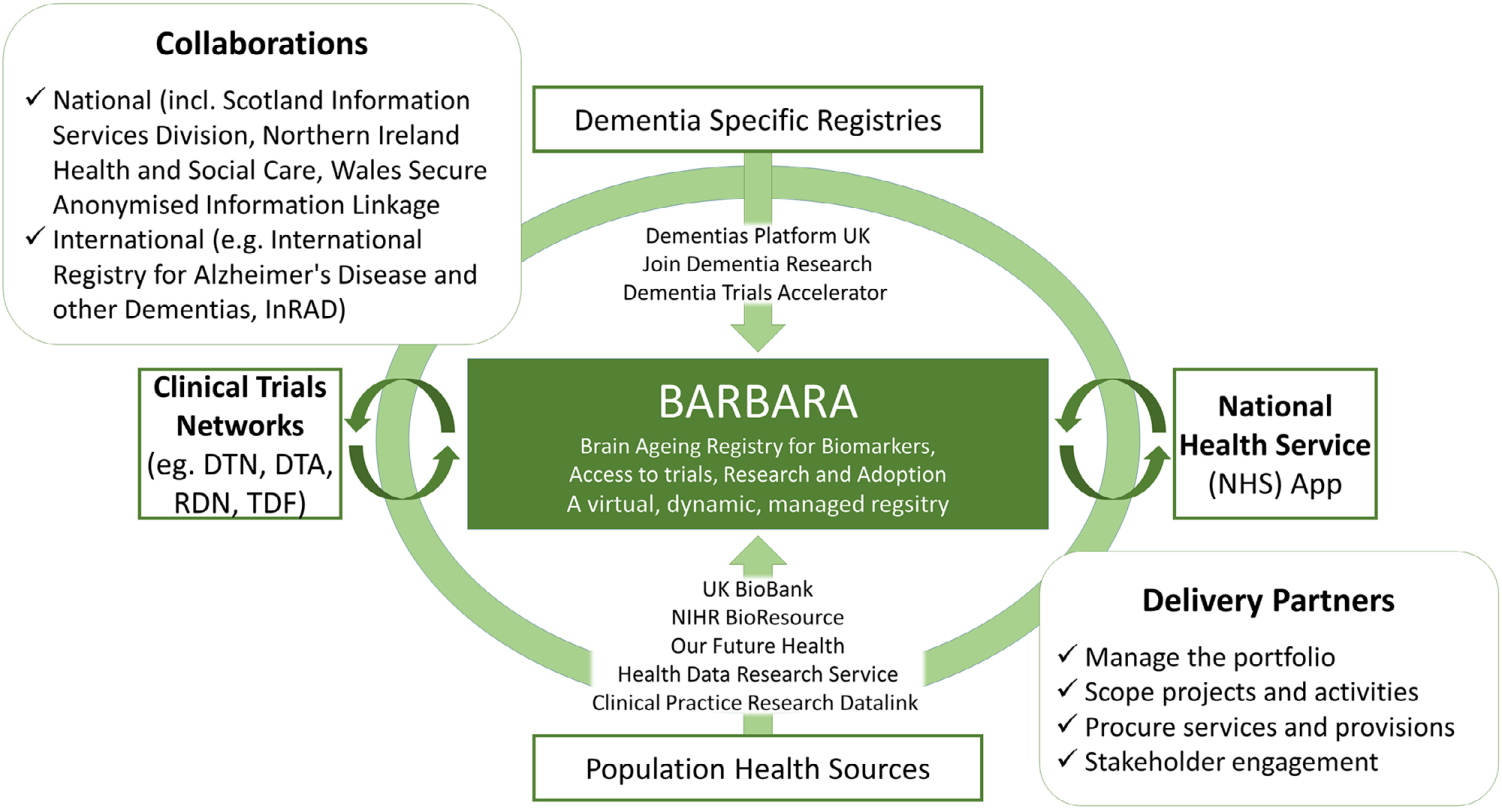
An illustrative model of how BARBARA will be deployed. BARBARA serves as a vehicle to consolidate its three strategic pillars. BARBARA will interact with the clinical trials ecosystem (e.g. NIHR Dementia Trials Network, NIHR research delivery networks, Dementia trials accelerator and UK Dementia Research Institute, DPUK trial delivery framework, join dementia research), and NHS app and patient‐facing tools, enabling dynamic data flow to support recruitment, patient engagement, and longitudinal monitoring. Additional inputs include dementia registries, UK initiatives, and population health resources that contribute structured, consented data to BARBARA, strengthening cohort development, real‐world evidence, and analytical capabilities. Delivery partners will manage the project portfolio, scope initiatives, and procure services. BARBARA will be open to integrate and collaborate with existing national and international initiatives, enabling data integration, shared research efforts, and alignment with broader dementia research infrastructures including the devolved nations (Scotland: ISD, Wales: SAIL, Northern Ireland: HSCNI) and international partners (e.g. InRAD). DPUK, Dementias Platform UK; NHS, National Health Service; UK, United Kingdom.

In sum, a living, inclusive infrastructure that enables the United Kingdom to become a global leader in dementia trials, supporting both academic and commercial research while prioritizing public trust, accessibility, and system readiness (See Figure 2).

BARBARA will be designed to:

- Enable patients to pre‐consent for future clinical trial opportunities and RWD use.
- Recruit from both at‐risk and diagnosed populations via structured, consented data from existing registries (e.g. DPUK, DTA JDR), population cohorts (e.g., OFH, UK Biobank, NIHR BioResource), and NHS records.
- Integrate with the NHS App and other patient‐facing tools to support engagement.
- Support longitudinal monitoring and stratification through biomarkers like pTau217 and digital cognitive assessments.

Crucially, BARBARA will support both research and implementation:

- For research: provide dynamic pre‐screened trial‐ready cohorts (e.g. UKDTN), reduce screen failure rate for sponsors; enable meta‐analyses across cohorts.
- For implementation: enable NHS‐based patient stratification, support evaluation of treatment effectiveness, and guide health technology assessments.

Its federated structure will allow data custodians (e.g., UK Biobank, Our Future Health, NIHR BioResource, JDR, etc.) to retain ownership, while contributing to a shared national resource with greater collective value. Data governance will follow FAIR principles (findable, accessible, interoperable, and reusable), ensuring transparency, interoperability, and trust.

BARBARA will also create a continuous learning loop: insights from trials and treatments will feed back into the registry, refining recruitment, improving diagnosis, and accelerating innovation. By reducing screen failure rates and pre‐identifying eligible participants and known cognitive status (whether unimpaired, asymptomatic‐prodromal, or symptomatic‐impaired), with biomarkers such as pTau217, BARBARA will significantly reduce trial costs and improve recruitment efficiency for commercial sponsors. In this context, pTau217 is considered an early marker of underlying AD pathology that can also track longitudinal changes over time. Importantly, a single pTau217 assay does not in itself identify individuals in progressive decline, but rather can detects early pathological changes. These changes may precede clinical symptoms, supporting participant stratification as well as monitoring through a trial or the disease course. All trial participants should be invited to consent to future data linkage at enrolment, enabling secure, longitudinal integration of clinical, research, and outcomes data.

Early engagement with regulatory authorities, including the Medicines & Healthcare products Regulatory Agency (MHRA), National Institute for Health and Care Excellence (NICE) and equivalent bodies in the devolved nations (e.g., the Scottish Medicines Consortium [SMC] and relevant NICE arrangements in Northern Ireland), is necessary to prepare for biomarker use, companion diagnostics, and RWD applications alignment with evidentiary standards and approval pathways. Once operating at scale, RWD from BARBARA could support regulatory submissions, adaptive licensing, and post‐market safety monitoring.

To succeed, BARBARA must be designed as an independent, professionally managed entity, that works in close collaboration with the NHS but remains operationally separate. This structure will enable greater flexibility in managing data, partnerships, and innovation, while avoiding the constraints of NHS infrastructure and procurement. Crucially, BARBARA must still align with clinical pathways to ensure relevance to real‐world care and support public trust.

Strong partnerships with patient groups, dementia charities, and community leaders will be essential to promote equitable participation and inclusion, particularly among underserved populations. The People's Forum, co‐chaired by Hilary Evans‐Newton and Scott Mitchell, was established to ensure that the Dementia Goals Programme is shaped by those with lived experience. Their input has grounded the programme in real‐world needs and defined priorities such as cultural relevance, accessibility, and trust. Ongoing collaboration with people with lived experience, through the Forum and leading dementia charities, will be essential to ensure BARBARA remains co‐designed, inclusive, and patient‐centered.

In summary, BARBARA will be the unifying national infrastructure to enable scalable, efficient, and inclusive dementia research and care, connecting patients, data, and trials through a secure, transparent, and adaptive ecosystem that can collaborate with existing national and international efforts, including Devolved Nations (e.g. ISD, SAIL, HSCNI) and global partners like InRAD. Notably, the virtual registry approach could be adapted for other major health conditions facing similar challenges in clinical trial recruitment and RWD integration, such as mental health, obesity, or other chronic diseases, by leveraging the same population health data, digital infrastructure, and existing registries.

## 4 BIOMARKERS FOR DEMENTIA: A STEPWISE APPROACH TO IMPLEMENTATION

Biomarkers are measurable indicators of biological processes, pathological changes, or responses to therapeutic interventions. They may include molecules, genes, enzymes, proteins, or digital cognitive performance metrics. The value of a biomarker becomes meaningful when paired with its intended use, such as risk prediction, diagnosis, treatment selection, or disease monitoring.^9^

The SAB recommends that biomarkers be integrated into the BARBARA registry in a phased manner, based on their context of use and validation status. This stepwise approach enables scalable implementation while preserving clinical utility and ensuring regulatory alignment.

### 4.1 Step 1: Identify at‐risk individuals

The initial focus should be on risk identification, particularly for Alzheimer's disease (AD), the most common form of dementia. The SAB recommends prioritizing two validated biomarkers, one BBM and one digital cognitive test to assess pathological change and functional decline, respectively.

We recommend the inclusion of plasma pTau217, a highly accurate indicator of presymptomatic amyloid pathology. Studies show strong associations with amyloid and tau status, cortical thickness, and cognitive decline.^10^, ^11^, ^12^ Recently approved by the United States Food and Drug Administration (US FDA) and in early use within UK research settings, pTau217 offers a scalable, non‐invasive alternative to positron emission tomography (PET) or lumbar puncture.

While not recommended as a core requirement for the registry, apolipoprotein E4 (APOE4) status is a well‐established genetic risk factor for AD and may inform stratification in certain contexts, particularly in trials involving amyloid‐targeting therapies.^13^ Many clinical trials include APOE4 status. Where this already exists, it is valuable to include it for research or trial‐enrollment purposes. However, its broader utility and cost‐effectiveness should be considered on a study‐by‐study basis rather than mandated for all participants.

We recommend pTau217 testing every 2–3 years, and more frequently (e.g. every 12–24 months) if symptomatic or with abnormal previous results, to assess increasing risk of the most common type of dementia in the presence of elevated amyloid and/or tau. A venous blood sample is currently required, but finger‐prick technology is advancing quickly and would facilitate use in home settings.^14^

Individuals with both normal pTau217 levels and preserved cognition at baseline would not be recruited into the initial BARBARA infrastructure, allowing the registry to focus on the needs for treatment of and secondary prevention trials. They could be recruited at a later date on repeat assessment (e.g. after 2–3 years). Blood‐based biomarkers (including pTau217) and digital or clinical cognitive measures therefore will serve both as initial inclusion and stratification tools and as longitudinal monitoring measures. This allows for reassessment over time, with individuals who initially present with normal biomarker and cognitive profiles to be included if their biomarker or health status changes.

In addition to biomarkers that report brain pathology, all participants should also complete a brief digital cognitive test battery via mobile phone or tablet. Partnerships with UK‐based commercial providers will be required to ensure availability, harmonization, and cultural relevance. Support mechanisms should be established to reduce digital exclusion, especially in underserved communities to ensure access to digital technologies.^15^

### 4.2 Step 2: Enable differential diagnosis

The second phase involves the development of a diagnostic biomarker toolbox to accurately diagnose and further understand the pathology of patients that have been confirmed at high risk. There is a significant opportunity to create a pathway for more accurate diagnosis, and this is currently underway through the DTA.

There are a myriad of currently available blood‐based and cerebrospinal fluid (CSF) biomarkers including markers of amyloid‐β and Tau pathology diagnosis, Aβ1‐42, Aβ42/40 ratio, pTau181, pTau217 and Total Tau.^16^, ^17^, ^18^, ^19^ A further set of neurodegeneration and inflammation markers including neurofilament light chain (NfL), and glial fibrillary acidic protein (GFAP)^10^, ^20^ are also used extensively in research, and diagnosis of other neurodegenerative disorders (e.g Parkinson's disease, amyotrophic lateral sclerosis [ALS], and Huntington's disease) would benefit from further differential blood based or digital biomarkers.

### 4.3 Step 3: Develop a unified diagnostic panel

As research advances, the field should transition from testing a single‐analyte to measuring a consolidated panel of BBMs that capture multiple pathologies and treatment‐relevant endpoints. UK researchers already have the capacity to measure > 100 components from a single plasma sample.

The SAB recommends funding the development, optimization, UKCA/CE marking, and commercialization of such a panel for use in subjects shown to be at significant risk. This work should be coordinated with digital cognitive tools and include age, genetic risk scores, and sociodemographic factors to improve predictive accuracy.

Parallel efforts are needed to integrate emerging passive digital tools, which can track behavior and cognitive decline without active testing. These may offer less intrusive alternatives for ongoing monitoring but must priorities inclusivity and accessibility.^21^ READ‐OUT has selected two digital cognitive test batteries as their prototypes assessments to act as benchmarks against which improvements can be assessed.

### 4.4 Regulatory considerations

Early engagement with the MHRA and other regulatory authorities is essential to align on:

- Validated use cases (e.g., risk stratification vs. companion diagnostics).
- Data and evidence requirements for clinical deployment.
- Standardization of biomarker thresholds and diagnostic criteria.

A phased, evidence‐driven approach to biomarker integration will ensure BARBARA delivers clinical, research, and regulatory value. The SAB recommends starting with pTau217 and digital cognitive testing, expanding to a differential diagnosis toolbox, and ultimately consolidating into a unified biomarker panel for further characterization of those shown to be at risk This strategy supports the overarching aim of BARBARA: to create a trusted, patient‐centered, scalable platform for dementia diagnosis, trial recruitment, and therapeutic monitoring.

The recommendations of the Scientific Advisory Board are closely aligned with the Government's 10‐Year Health Plan and Life Sciences Sector Plan,^22^ supporting priorities such as earlier diagnosis and prevention, reducing health inequalities through inclusive research access, and embedding digital and biomarker‐based innovations into routine care to enable more personalized and equitable dementia services.

## 5 IMPLEMENTATION PLAN

As with all initiatives, the challenge is not in seeing what could be done but in implementation. The SAB has given some thought to the first key first steps which will need to be developed and expanded much further by the organization responsible for the BARBARA registry. Transitioning from the current fragmented ecosystem to a coherent, virtual national registry of willing, well‐characterized individuals will take time, but the technology, need, and momentum already exist. Multiple workstreams must begin concurrently to ensure timely delivery.

### 5.1 Identify an independent delivery organization

Identify an independent organization to manage the BARBARA registry. A model similar to Genomics England would balance public accountability with operational flexibility and commercial partnership potential. This structure (eithera.comora.org) is essential for strong governance, trust‐building, and long‐term sustainability and a leader should be appointed as a first step.

### 5.2 Define governance and data standards

Implement robust governance structures that ensure secure, ethical access to patient‐level data, supported by appropriate consent processes. The BARBARA registry must adhere to FAIR principles to enable integration with NHS systems and national data environments.

### 5.3 Validate biomarkers in NHS settings

Advance further real‐world validation of selected blood‐based and digital biomarkers through ongoing studies such as READ‐OUT, Bio‐Hermes 2, and the DTA. These studies will generate the evidence needed for adoption into NHS workflows and future BARBARA enrolment criteria.

### 5.4 Standardize tools for broad use

Tender for validated platforms (with an acceptable path to validation by MHRA) to measure pTau217 and deliver digital cognitive assessments ensuring consistency across participating sites. These tools will serve as the initial inclusion criteria for individuals entering the registry.

### 5.5 Enable integration of existing cohorts

Build systems to allow participants from existing studies (e.g., Our Future Health, UK Biobank, JDR) to opt into BARBARA without re‐enrolment, using harmonized consent and recall mechanisms. This federated model respects data sovereignty while improving recontact for clinical trials and care.

### 5.6 Engage regulators early

Work proactively with the MHRA (Innovative Devices team within Standard and Compliance) and other regulatory bodies to define approval pathways for biomarkers used in diagnosis, stratification, and therapeutic monitoring. Early alignment on evidence standards will prevent delays and support broader NHS adoption.

### 5.7 Refine and expand the data model

Build from an initial two‐biomarker inclusion approach (pTau217 + digital cognition) toward a more comprehensive diagnostic panel, incorporating genetic, phenotypic, and longitudinal data. This will enhance precision and enable alignment with international biomarker frameworks.

### 5.8 Implement a national communication and engagement strategy

Raise awareness of the registry through an inclusive, multi‐channel strategy co‐developed with the People's Forum. Engagement should priorities underrepresented communities and provide culturally appropriate, multilingual materials delivered by trusted voices (e.g., general practitioners [GPs], community leaders, individuals living with dementia). Educational initiatives should be coordinated with existing programs to avoid duplication and increase reach.

## 6 CONCLUSION

Dementia is one of the most pressing global health challenges, yet progress in developing effective treatments has been hindered by low clinical trial participation, particularly among underrepresented populations. The Dementia Goals Programme was established to address this gap, and the SAB recommends the creation of a nationwide virtual registry to support the development, evaluation, and equitable deployment of new treatments.

The BARBARA registry will streamline recruitment, support diverse participation, and enable faster access to innovative therapies across the United Kingdom. It will also provide RWD to inform trial design, regulatory decisions, and long‐term monitoring, thereby improving our understanding of the disease and accelerating progress toward personalized care.

While key questions remain and require further research investment, the creation of a dynamic registry built on standardized biomarkers offers a blueprint not only for dementia, but potentially for other complex disorders. For dementia specifically, the long and variable disease course, heterogeneity of pathology, and urgent unmet need demand a coordinated national response.

The future of dementia care depends on the action we take now. BARBARA represents a bold step toward a more inclusive, efficient, and effective research and care ecosystem.

## CONFLICT OF INTEREST STATEMENT

J.B.R. has provided consultancy to Alector, Asceneuron, Astronautx, Alzheimers Research UK, Astex, Aviado Bio, Booster Therapeutics, Boehringer Ingelheim, ClinicalInk, Curasen Therapeutics, Cumulus Neuro, Eisai, Ferrer, SV Health, Prevail, Vesper Bio, and UCB; and received research grants from AstraZeneca, Lilly, GSK, and Janssen as part of the Dementias Platform UK and is Chief Scientific Advisor to Alzheimers Research UK. C.E.T. has research contracts with Acumen, ADx Neurosciences, AC‐Immune, Alamar, Aribio, Axon Neurosciences, Beckman‐Coulter, BioConnect, Bioorchestra, Brainstorm Therapeutics, C2N diagnostics, Celgene, Cognition Therapeutics, EIP Pharma, Eisai, Eli Lilly, Fujirebio, Instant Nano Biosensors, Merck, Muna, Nitrase Therapeutics, Novo Nordisk, Olink, PeopleBio, Quanterix, Roche, Sysmex, Toyama, Vaccinex, and Vivoryon. She has consultancy/speaker contracts for Aribio, Biogen, Beckman‐Coulter, Cognition Therapeutics, Danaher, Eisai, Eli Lilly, Janssen, Merck, Neurogen Biomarking, Nordic Biosciences, Novo Nordisk, Novartis, Olink, Quanterix, Roche, Sanofi, and Veravas. J.H. has consulted for Eisai and is honorary VP for ARUK. S.P.S. has provided consultancy to Alzheimers Research UK. She is specialist advisor to the NIHR RSS delivered by Imperial College London and Parters, and received research grants from NIHR i4i, NIHR PDG, and UKRI NERC, which focused on the environmental impact on health, and spatial biomarkers of early Alzheimer's disease. R.M. is an operating partner for SV Healthinvestors and the Dementia Discovery Fund, chair of Cumulus Neuroscience, Board member of AstronauTx and ARUK, and a scientific advisor to Biogen. Oher authors declare no conflicts. Author disclosures are available in the Supporting Information.

## Supporting information



Supporting Information

Supporting Information

Supporting Information
