# Supplementary material for: Breaking barriers: Enhancing access to dementia clinical trials in the United Kingdom—Insights from the Scientific Advisory Board of the Dame Barbara Windsor Dementia Goals Programme
Source: Alzheimers Dement. 2026 Jul 5;22(7):e71621. doi: 10.1002/alz.71621 (PMC13333077; doi:10.1002/alz.71621)
Supplement: Supplementary file 1 — Supporting Information [file ALZ-22-e71621-s002.docx]

**Appendix.** Summary of Key Current and Emerging Dementia Registries in the UK and Internationally: The table provides an overview of existing and potential large-scale registries relevant to dementia research. It highlights their scope, scale, and functionality, ranging from UK-based clinical and research registries to international initiatives.
