# Supplementary material for: Breaking barriers: Enhancing access to dementia clinical trials in the United Kingdom—Insights from the Scientific Advisory Board of the Dame Barbara Windsor Dementia Goals Programme
Source: Alzheimers Dement. 2026 Jul 5;22(7):e71621. doi: 10.1002/alz.71621 (PMC13333077; doi:10.1002/alz.71621)
Supplement: Supplementary file 2 — Supporting Information [file ALZ-22-e71621-s001.docx]

**Glossary**

**BioHermes** – A research initiative led by Global Alzheimer's Platform Foundation (GAP) focused on improving Alzheimer's diagnosis through blood-based and digital biomarkers.[Bio-Hermes – Global Alzheimer’s Platform Foundation](https://globalalzplatform.org/biohermesstudy/)

**UK Biobank** – Large national cohort with extensive health/genetic data for diverse disease research. [UK Biobank - UK Biobank](https://www.ukbiobank.ac.uk/)

**CPRD-** Clinical Practice Research Datalink is a real-world research service supporting retrospective and prospective public health and clinical studies. CPRD research data services are delivered by the [Medicines and Healthcare products Regulatory Agency](https://www.gov.uk/government/organisations/medicines-and-healthcare-products-regulatory-agency/) with support from the [National Institute for Health and Care Research (NIHR)](http://www.nihr.ac.uk/), as part of the Department of Health and Social Care. [Clinical Practice Research Datalink | CPRD](https://www.cprd.com/)

**UK-DTN** – Dementia Translational Research Collaboration Trials Network: advancing dementia research through clinical trials, comprises a group of research active dementia themes within the national network of NIHR Biomedical Research Centres, along with CRFs and members of the devolved nations. [Dementia TRC | NIHR](https://www.nihr.ac.uk/about-us/what-we-do/infrastructure/translational-research-collaborations/dementia)

**Dementias Platform UK (DPUK) Clinical Studies Register** – A register of >58,000 pre-characterised participants with consent for re-contact. [Welcome — DPUK](https://www.dementiasplatform.uk/)

**DPUK Trials Delivery Framework-** Its aim is to bring the right people to the right place for the right project.This precision-matching of volunteers to early-phase trials based on detailed genetic and observed characteristics will ensure that researchers testing therapies and interventions can understand quickly and robustly what works – and what doesn't. Study participants will be recruited both from pre-clinical registries and through clinical referrals.[Trials Delivery Framework — DPUK](https://www.dementiasplatform.uk/research-hub/trials-delivery-framework)

**DTA** – Dementia Trials Accelerator: initiative to accelerate development and testing of new dementia treatments. It is funded by the Medical Research Council (MRC) and supports the UK government's Dame Barbara Windsor Dementia Goals program. [Dementia Trials Accelerator (DTA) | UK DRI](https://www.ukdri.ac.uk/dementia-trials-accelerator#:~:text=Spearheaded%20by%20the%20UK%20DRI,part%20of%20a%20clinical%20trial.)

**HDRS-** Health Data Research Service is is a major new initiative from the UK Government designed to make the UK the global leader in medical research. Announced by the Prime Minister as part of a broader Plan for Change and modern industrial strategy, HDRS will create a secure, centralised access point for NHS data, streamlining how researchers access national-scale datasets. [Prime Minister turbocharges medical research - GOV.UK](https://www.gov.uk/government/news/prime-minister-turbocharges-medical-research)

**HSCNI-** Health and Social Care Northern Ireland is the publicly funded healthcare system in Northern Ireland. Although having been created separately to the National Health Service, it is nonetheless considered a part of the overall national health service in the United Kingdom. [hscni.net | hscni.net](https://www.hscni.net/)

**InRAD-** International Registry for Alzheimer’s Disease and Other Dementias is a free registry that helps with the collection, combination, and comparison of real-word data. The registry is coordinated by the independent International Registry for Alzheimer’s Disease and Other Dementias Foundation, a health-related not-for-profit entity incorporated in the Netherlands. [Home](https://www.inradnetwork.org/)

**ISD-** Information Service Division is a standard set of aggregated summary statistics on activity in hospitals and other health care settings in Scotland. ISD provides health information, health intelligence, statistical services and advice that support the NHS in progressing quality improvement in health and care and facilitates robust planning and decision making. [What is ISD(S)1? - ISD(S)1 hospital activity - Data management in secondary care: hospital activity - Health intelligence and data management - Resources and tools - Public Health Scotland](https://publichealthscotland.scot/resources-and-tools/health-intelligence-and-data-management/data-management-in-secondary-care-hospital-activity/isd-s-1-hospital-activity/what-is-isd-s-1/)

**Join Dementia Research (NIHR JDR)** – UK registry connecting volunteers with dementia studies. [Join dementia research - register your interest in dementia research : Home](https://www.joindementiaresearch.nihr.ac.uk/)

**MHRA-** Medicines and Healthcare products Regulatory Agency regulates medicines, medical devices and blood components for transfusion in the UK. MHRA is an executive agency, sponsored by the Department of Health and Social Care. [Medicines and Healthcare products Regulatory Agency - GOV.UK](https://www.gov.uk/government/organisations/medicines-and-healthcare-products-regulatory-agency)

**NICE-** National Institute for Health and Care and Excellence it helps practitioners and commissioners get the best care to patients, fast, while ensuring value for the taxpayer. [Homepage | NICE](https://www.nice.org.uk/)

**NIHR BioResource** – Volunteer panel for research based on genetic and health data. [Join the BioResource](https://www.bioresource.nihr.ac.uk/participants/join-the-bioresource/)

**OFH-** Our Future Health is an ambitious collaboration between the public, charity and private sectors to build the UK’s largest health research programme – bringing people together to develop new ways to prevent, detect and treat disease. [About us – Our Future Health](https://ourfuturehealth.org.uk/about-us/)

**Primary Care Dementia Data Series-** the largest UK collection of data points representing individuals with dementia diagnoses within a primary care setting, aggregating monthly information on MCI and dementia diagnoses (incidence and prevalence) from every GP surgery in England at locality level. [Primary Care Dementia Data - NHS England Digital](https://digital.nhs.uk/data-and-information/publications/statistical/primary-care-dementia-data)

**READ-OUT** – REAl World Dementia OUTcomes: part of the Blood Biomarker Challenge led by Prof. Vanessa Raymont, which aims to capitalise on recent breakthroughs in potential dementia blood tests, and generate the evidence needed for them to be validated for use in the NHS within the next 5 years. [READ-OUT — DPUK](https://www.dementiasplatform.uk/research/read_out)

**SAIL-** Secure Anonymised Information Linkage, provides a rich and trusted data resource, exceptional in many respects, supporting research for patient and public benefit: a vital facility for interdisciplinary team science. [Home - SAIL Databank](https://saildatabank.com/)
